# Deep Learning-Based Congestion Detection at Urban Intersections

**DOI:** 10.3390/s21062052

**Published:** 2021-03-15

**Authors:** Xinghai Yang, Fengjiao Wang, Zhiquan Bai, Feifei Xun, Yulin Zhang, Xiuyang Zhao

**Affiliations:** 1 School of Information Science and Technology, Qingdao University of Science and Technology, Qingdao 266061, China; 2 School of Information Science and Engineering, University of Jinan, Jinan 250022, China; 201821200705@mail.ujn.edu.cn (F.W.); 2016220568@mail.ujn.edu.cn (F.X.); ise_zhangyl@ujn.edu.cn (Y.Z.); ise_zhaoxy@ujn.edu.cn (X.Z.); 3 School of Information Science and Engineering, Shandong University, Qingdao 266237, China

**Keywords:** congestion detection, image processing, optical flow, surveillance video, YOLOv3

## Abstract

In this paper, a deep learning-based traffic state discrimination method is proposed to detect traffic congestion at urban intersections. The detection algorithm includes two parts, global speed detection and a traffic state discrimination algorithm. Firstly, the region of interest (ROI) is selected as the road intersection from the input image of the You Only Look Once (YOLO) v3 object detection algorithm for vehicle target detection. The Lucas-Kanade (LK) optical flow method is employed to calculate the vehicle speed. Then, the corresponding intersection state can be obtained based on the vehicle speed and the discrimination algorithm. The detection of the vehicle takes the position information obtained by YOLOv3 as the input of the LK optical flow algorithm and forms an optical flow vector to complete the vehicle speed detection. Experimental results show that the detection algorithm can detect the vehicle speed and traffic state discrimination method can judge the traffic state accurately, which has a strong anti-interference ability and meets the practical application requirements.

## 1. Introduction

With the significant increase of motor vehicles, traffic congestion is recognized as a serious problem globally. Reliance on widening the road areas to alleviate the traffic congestion has become more and more difficult. The focus on traffic congestion has turned to detection and facilitation. The traditional manual congestion detection method cannot completely cover the entire section of the road and thus causes problems, such as missed detection and delayed facilitation. Automatic congestion detection can make up for the lack of manual detection and improve the efficiency of congestion detection.

The urban traffic management evaluation index system issued by the Ministry of Public Security in 2002 [1] stipulates that the average travel speed of motor vehicles on urban trunk roads is used to describe the road traffic operation. Congestion happens when the speed of the vehicle becomes less than 30 km/h or it fails to pass through the intersection within three traffic light cycles [2]. Congestion determination indicators include traffic flow, vehicle speed, density, and occupancy [3]. In this study, the vehicle speed is used as the determination indicator combined with multiple detections in a signal light cycle, and its effectiveness in determining the traffic conditions is verified.

Traffic state discrimination algorithms have been divided into several kinds, such as pattern recognition, catastrophe theory, and statistical prediction. The most famous pattern recognition algorithm is the California algorithm with the key idea to compare the upstream and downstream occupancy of a road section. However, its accuracy is not high since the traffic flow and the vehicle speed are not taken into account [4,5] combined the background difference and optical flow methods to obtain the vehicle speed and direction information, and then employed the mean shift method to cluster the motion features and determine the behavior, but this method is more affected by noise [6]. Wei reduced the amount of detection calculations needed and realized the real-time traffic congestion detection through image texture analysis in [7]. A novel stereo vision-based system for vehicle speed measurement was proposed in [8], which was set in a fixed location to capture two-view stereo videos of the passing vehicles by a calibrated binocular stereovision system. [9] proposed a method on the basis of multisource global positioning system (GPS) data, which takes the k-means algorithm to cluster the data, obtain the average speed within the cluster, and determine the traffic congestion state.

A new sensing device [10] based on the combination of passive infrared sensors and ultrasonic rangefinders was developed for real-time vehicle detection, classification, and speed estimation in the context of wireless sensor networks [11]. The pattern recognition method can distinguish the traffic state quickly but requires a considerable number of samples to sum up the discriminants. However, a discriminant with a remarkable error is meaningless. Reference [12] presented the McMaster algorithm, which was based on the catastrophe theory, and added the two parameters, traffic flow, and vehicle speed, according to the original occupancy. The congestion state was modeled according to these three parameters to obtain results. Mutation theory considers occasional congestion and improves the accuracy of discrimination, but the adaptability of the algorithm is poor. Ke proposed a robust traffic flow parameter estimation method that is developed based on optical flow and traffic flow theory. Traffic flow parameter estimations in both free flow and congested traffic conditions are evaluated to obtain the final traffic state detection results in [13]. However, the calculation is complicated and time-consuming. For neural network methods, Henri established road scene and vehicle models to analyze the traffic flow in a specific scene and identified the traffic conditions in [14]. A method of selecting the evident features on the license plate area and tracking multiple frames to measure the vehicle speed was proposed in [15]. The neural network [16] has the ability to self-learning and can detect the vehicle target. However, it does not achieve satisfactory results between detection speed and accuracy. With the development of deep learning technologies, many researchers introduced deep learning algorithms [17] into the field of target detection and congestion detection in recent years. In order to achieve better accuracy, these algorithms need to constantly deepen the network, which may lead to huge calculation complexity. Therefore, this study proposes a new method that utilizes the four vertices of the vehicle target frame detected by YOLOv3 in [18] as the tracking points of the LK optical flow method [19], which greatly simplifies the calculation. At the same time, in the process of congestion detection, compare the global vehicle speed with the congestion threshold multiple times in a signal period to get the final traffic state. It is found that the judgment result of the proposed method is relatively more accurate than the typical congestion detection methods, such as Kernel fuzzy c-means clustering (KFCM) algorithm and Bayesian classifier.

The main contributions of this study are as follows:

(1) The LK optical flow method is improved by combining with the YOLOv3 algorithm to determine the exact position of the current vehicle and calculate the vehicle speed, which reduces the calculation of the optical flow value and achieves the real time performance without accuracy loss.
(2) A new traffic congestion detection algorithm has been proposed to judge the congestion state more accurately by comparing the vehicle speed with the congestion speed threshold within a signal period. Compared with the other congestion discrimination methods, such as the KFCM algorithm, a Bayesian classifier is generated by learning historical data of congestion state. The proposed traffic congestion detection algorithm may improve the discrimination accuracy of the congestion state from the slow driving state and is more feasible.

The remainder of this paper is organized as follows: Section 2 introduces the related work. Section 3 explains the YOLOv3 target detection algorithm and the LK optical flow method. The optical flow method integrated with the YOLOv3 for the vehicle speed measurement and the new traffic discrimination algorithm are proposed in Section 4. Section 5 provides the simulation results of the proposed new algorithm and verifies its efficiency and accuracy by comparisons with the other typical algorithms. Section 6 concludes the paper.

## 2. Related Work

This section analyzes the related work of urban intersection congestion detection. Most of the related researches on traffic state discrimination [20,21] were based on the single parameter evaluation, and the detection results are not accurate. With the development of artificial neural networks [22], the deep learning model is introduced into congestion detection to detect vehicle targets. However, it needs to build a deeper network to improve the detection accuracy, and the computational complexity is greatly increased, which is infeasible for real-time target detection. The YOLOv3 algorithm adopts the Resnet network structure to realize the cross-level connection and improve the detection speed without loss of accuracy [23]. Therefore, we focus on vehicle target detection with the application of the YOLOv3 algorithm and LK algorithm in this paper. We also compare our scheme with the latest popular and ideal detection algorithms, such as faster region CNN (faster R-CNN) algorithm and single shot multibox detector (SSD) algorithm. First of all, the vehicle data set obtained by video frame capture and crawler, then use the above three algorithms to train the vehicle dataset and compare the detection results. We found that the YOLOv3 algorithm achieves a good trade-off between the speed and accuracy of the vehicle target detection, even for small target detection. Meanwhile, the LK algorithm needs to track feature points to calculate vehicle speed, where the feature points are usually obtained by corner detection algorithms, such as Harris corner detection algorithm [24], Shi-Tomasi corner detection algorithm [25], and the other algorithms. In this paper, the vehicle target position detected by YOLOv3 is used as the corner of the LK algorithm for subsequent optical flow tracking, which simplifies the calculation of the corner detection algorithm. In the process of vehicle speed detection, we combine the YOLOv3 algorithm with optical flow method, traditional double coil virtual algorithm, and SSD target detection algorithm, and carry out the experiments in different conditions. It is shown that the proposed algorithm meets the requirements in speed and accuracy well, and has better adaptability to the environment.

A new traffic congestion detection algorithm has also been proposed in this paper. Since the previous congestion detection algorithms only take a specific indicator to determine the congestion at a certain point in time, their accuracy is not very good. In this work, we compare the speed obtained by the above algorithm in a traffic light cycle many times and divides the traffic state into smooth, slow driving and congestion to avoid misjudgment. After referring to the literature and observing the practical intersection traffic, we set the speed threshold of the congestion judgment as 30 km/h and carried out the experiments. It is found that this threshold gets good performance and accuracy. Also, compared with the KFCM algorithm [26] and Bayesian algorithm [27] commonly used in the field of congestion judgment, the proposed algorithm can detect the current road state more accurately.

## 3. YOLOV3 Target Detection and Optical Flow Tracking Method

### 3.1. YOLOv3 Target Detection Algorithm and Optical Flow Tracking Method

**Figure 1:**
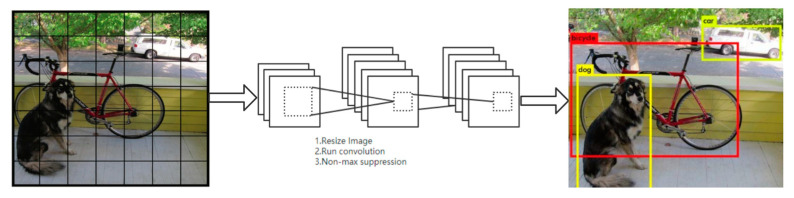
Schematic diagram of YOLO target detection.

YOLOv3 is an end-to-end algorithm that can speed up the detection and does not produce candidate frames. Figure 1 shows the target detection diagram of YOLO in GitHub [28]. We employ it to explain the process of target detection.

YOLOv3 divides the picture into S × S grids, and each grid detects the target located at the corresponding center. Each grid has three anchor boxes to predict the three target bounding boxes. The size and position for each target bounding box can be characterized by a data set (*x*, *y*, *w*, *h*, *s*), where (*x*, *y*) represents the central coordinate of the target bounding box relative to the grid, w and h are the width and height of the target bounding box, respectively, and *s* is the confidence score that reflects the possibility of the target contained in the target bounding box. We can write *s_i_* as,

(1) $$s_{i}=P_{r}(class_{i}|object)*P_{r}(object)*IOU_{pred}^{truth}=P_{r}(class_{i})*IOU_{pred}^{truth}$$

where *P_r_* represents the possibility of the objects in the predicted bounding box of the current mesh, and *IOU* (intersection and union ratio) denotes the accuracy of the predicted position of the target bounding box. *IOU* can be expressed as:

(2) $$IOU_{pred}^{truth}=\frac{box_{t}\cap box_{p}}{box_{t}\cup box_{p}}$$

where *t* is the real target border, *p* is the predicted border, *box_t_* represents the real target border in the image, and *box_p_* means the predicted target border.

YOLOv3 employs darknet-53 to extract the features. Darknet-53 has a considerable number of the residual skip layers and can converge when the network is built very deep. Therefore, YOLOv3 can get much better features to make the detection results more accurate compared with the YOLOv2 in [29].

### 3.2. LK Optical Flow Algorithm

The basic principle of the optical flow method is that the object in the image can be regarded as the same object if the pixel grayscale does not change in any two adjacent frames and the adjacent pixels have the same moving direction.

For example, the gray level of a pixel in the first frame is *f*(*x*, *y*, *t*), which will have a movement (*dx*, *dy*) in the next frame after time *dt*. The pixel point is considered as the same if the following equation is met:

(3) f(x,y,t)=f(x+dx,y+dy,t+dt)

We may take Taylor expansion to the right of (3), and the similar terms can be combined and divided by *dt* to obtain the optical flow equation as:

(4) $$f_{x}u+f_{y}v+f_{t}=0$$

where:

(5) $$f_{x}=\frac{\partial f}{\partial x};f_{y}=\frac{\partial f}{\partial y};u=\frac{dx}{dt};v=\frac{dy}{dt}$$

where *μ* and *v* represent the moving components of the optical flow in horizontal and vertical directions, respectively.

The LK optical flow method introduces a window with the target point *P* as the center and calculates the points *q*_1_, *q*_2_, …, *q_n_* in the window. We have the following equations:

(6) $$f_{x}(q_{1})u+f_{y}(q_{1})v=-f_{t}(q_{1})$$

(7) $$f_{x}(q_{2})u+f_{y}(q_{2})v=-f_{t}(q_{2})$$

⋮

(8) $$f_{x}(q_{n})u+f_{y}(q_{n})v=-f_{t}(q_{n})$$

which can be further expressed as **A**^T^**Av** = **A**^T^**b** with:

(9) $$A=\left[\begin{array}{cc} f_{x}(q_{1}) & f_{y}(q_{1})\\ \begin{array}{c} f_{x}(q_{2})\\ \vdots \\ f_{x}(q_{n}) \end{array} & \begin{array}{c} f_{y}(q_{2})\\ \vdots \\ f_{y}(q_{n}) \end{array} \end{array}\right]$$

(10) $$v=\left[\begin{array}{c} u\\ v \end{array}\right]$$

(11) $$b=\left[\begin{array}{c} -f_{t}(q_{1})\\ -f_{t}(q_{2})\\ \vdots \\ -f_{t}(q_{n}) \end{array}\right]$$

and we have **v** = (**A**^T^**A**)^−1^**A**^T^**b**. The matrix form representation can be also written as:

(12) $$\left[\begin{array}{c} u\\ v \end{array}\right]={\left[\begin{array}{cc} \sum_{i}f_{x}{\left(q_{i}\right)}^{2} & \sum_{i}f_{x}(q_{i})f_{y}(q_{i})\\ \sum_{i}f_{x}(q_{i})f_{y}(q_{i}) & \sum_{i}f_{y}{\left(q_{i}\right)}^{2} \end{array}\right]}^{-1}\left[\begin{array}{c} -\sum_{i}f_{x}(q_{i})f_{t}(q_{i})\\ -\sum_{i}f_{y}(q_{i})f_{t}(q_{i}) \end{array}\right]$$

Finally, we can obtain the motion field of the window can to determine whether the object is the same to complete the tracking of the target.

## 4. Improved Global Speed Detection and Traffic State Discrimination Algorithm

This section proposes the congestion detection scheme which is divided into two parts, namely, global speed detection algorithm and traffic state discrimination algorithm. The former obtains the region of interest (ROI) according to the framing characteristics of the urban intersection monitoring. ROI aims to outline the area to be processed in the target image marked in the form of box, circle, ellipse, irregular polygon, and so on. The YOLOv3 algorithm is used to detect the vehicle target combined with LK optical flow method, aiming to calculate the optical flow to obtain the global vehicle speed. Thus, the traffic state discrimination algorithm can analyze global vehicle speed to obtain a real time traffic state.

### 4.1. Global Speed Detection Algorithm

**Figure 2:**
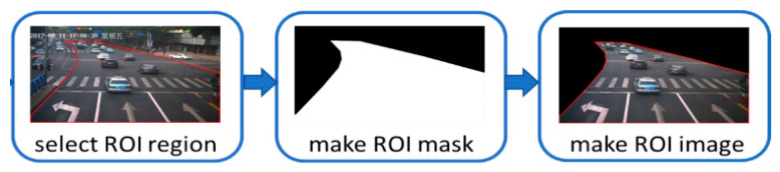
ROI image acquisition.

**Figure 3:**
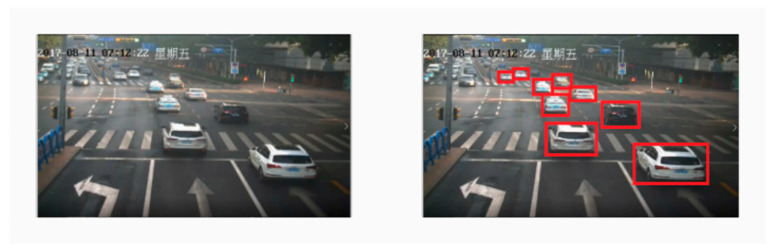
YOLOv3 target detection results.

The ROI is selected by clipping the whole picture to improve the image processing speed and the image recognition accuracy. A camera is set to capture the information of the current lane in the forward lane direction. Therefore, each camera only needs to pay attention to the picture of the lane on its side and complete the comprehensive monitoring of the intersection road. The ROI mask is obtained based on the selected ROI and it is a binary image of the same size as the original image. ROI image acquisition process is shown in Figure 2, where the data comes from the intersection of Jingshi Road (Jinan City, China), and is taken from a project named “Analysis of Jinan Traffic” in our group. The vehicles in the ROI are detected with the YOLOv3 algorithm. Figure 3 illustrates the detection results also based on the data from Figure 2. In the following, we use the same data obtained from our project.

The YOLOv3 algorithm is able to detect the vehicle targets accurately, even some small targets within the distance. The four vertexes of the detection frame (the red boxes on the right side of Figure 3) obtained by YOLOv3 were used as the optical flow input to track and get the vehicle speed. We can measure the global vehicle speed with this method. When YOLOv3 loses one or two targets or the individual vertexes of the output detection frame, our global average vehicle speed detection will not be affected. Algorithm 1 shows the improved algorithm.

**Figure 4:**
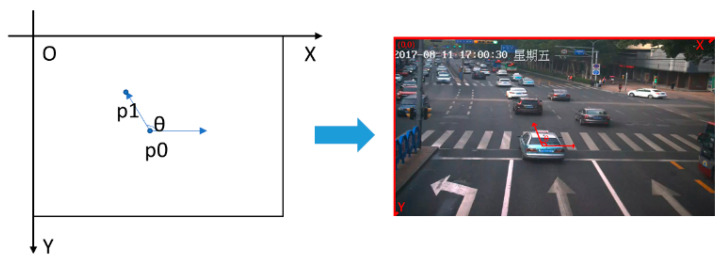
Construction of coordinate system and calculation of the included angle.

**Algorithm 1** Global Speed Detection Algorithm.Input: Intersection monitoring of ROI, YOLOv3 detection box vertex p0, optical flow error threshold Td, traffic direction Oi, and video frame rate N.Output: Global vehicle speed V.1. The vertex of the bounding box output by YOLOv3 is the feature point *p*_0_ and the number of the feature points is *n*.2. LK optical flow method is used to calculate the next frame’s position (from p_i_ to *p_i_*_+ 1_); then, the same method is used to calculate the position of *p_i_*_+ 1_ in the previous frame, denoted as *p*_ir_.3. For *i* = 1; *i* ≤ *n*; *i*++do.4.        if *d* = |*p_i_* − *p_ir_*| < *T_d_* then:5.            retain *p_i_*;6.        else:7.            discard *p_i_*.8.        As shown in Figure 4, to construct a coordinate, computing the angle *θ* with respect to $\vec{P_{i}P_{i+1}}$ and X axis to get the vehicle direction *O_i_*.9.        If 90° ≤ *θ* ≤ 140°, then:10.            reserve the corner point;11.        else:12.            discard the corner point.13. Compute the mean value of *N* frame speed as global vehicle speed *V* and output it.

In this paper, the optical flow method is employed, since it can accurately identify the target location without the information of the scene, which is more suitable for the congestion detection, especially for the dense and rapidly changing scenes of vehicles. The dense optical flow is used to calculate the optical flow value for each pixel in the image, but the amount of the calculation is extremely large. In this study, the optical flow method is combined with YOLOv3 target detection, and the four vertices of the output detection frame of the YOLOv3 algorithm are taken as the input to the optical flow method for optical flow calculation, which minimizes data processing while ensuring the integrity of the image features. The experimental image size in this paper is set as 1280 × 720. Tracking the vertex of the YOLOv3 detection frame makes the object of the optical flow calculation drop from 100,000 order of magnitude to several feature points. Therefore, the amount of the calculation can be greatly reduced. At the same time, the velocity is obtained directly by calculating the optical flow value, which reduces the algorithm complexity without converting the two-dimensional coordinate system to the spatial coordinate system [30] and meets the real-time requirements.

### 4.2. Traffic State Discrimination Algorithm

**Figure 5:**
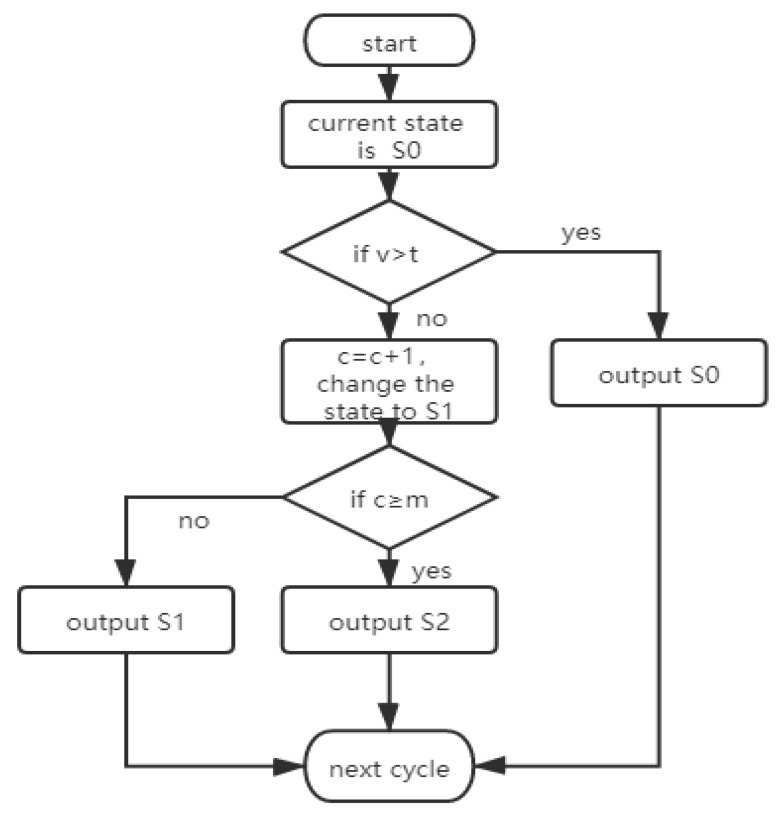
Traffic state recognition algorithm.

The speed obtained from the global speed algorithm in Section 3.1 is further used as the parameter to distinguish the traffic congestion. In this subsection, we divide the traffic condition into three states: congestion (vehicle detention affects the traffic travel), slow driving (vehicle detention occasionally exists but does not affect the travel), and smooth traffic (no vehicle detention and it does not affect the travel). Vehicle speed will be very low during the red-light waiting time and congestion state and thus makes the detection results difficult to distinguish. Therefore, we can judge the vehicle speed value to determine the presence of congestion, select a signal light cycle for multiple continuous speed discrimination, and determine the final traffic state as shown in Figure 5.

First, we define the following three congestion states, non-congestion state (*S*_0_), suspected congestion state (*S*_1_), and final congestion state (*S*_2_). *v* is the average speed of I seconds, *t* denotes the congestion threshold, *c* is the current number of judgments, and *m* is the maximum number of congestion judgments. The output traffic state will result in slow driving when the vehicle enters the congestion state *S*_1_ if *c* < *m* and *v* < *t*. *S*_1_ is suspected to turn to be congestion state *S*_2_ once *c* ≥ *m* and the output of the traffic state results is congestion. Algorithm 2 shows the detailed steps.

**Algorithm 2** Traffic State Discrimination Algorithm.**Input**: Average the vehicle speed *v* (in I seconds), congestion speed threshold *t*, and maximum times of the congestion judgment *m***Output**: Traffic state S Starting at *S*_0_, set *c* = 0.for *i* = 1; *i* ≤ *m*; *i*++doif *v_i_* > *t* then:stay at *S*_0_, and output non-congestion signal, smooth state;else:*c* = *c*+1 change the state to *S*_1_.if *c* < *m*, then:output state is slow;else:change the state to *S*_2_, output congestion signal.

## 5. Experiments and Result Analysis

In this subsection, we utilize the proposed algorithm to judge the traffic conditions. Given the video of the road intersection, we first use the YOLOv3 algorithm to detect the vehicle target of the intersection, according to the global vehicle detection method. Then, the improved LK optical flow method is employed to calculate the vehicle’s optical flow value to obtain the global speed. Finally, based on the traffic state discrimination algorithm, the traffic state can be achieved. In order to illustrate the advantages of the proposed algorithm, we compare it with the classical and the traffic state discrimination algorithms in the following.

The speed measurement is based on the speed formula according to the displacement of the vehicle and the frame rate of the video. The popular methods are the traditional speed detection based on the virtual coil and the current speed detection based on the feature matching. The virtual coil speed detection based on the double detection lines fixes the distance between two detection lines and gets the real running speed by calculating the time that the vehicle passes the distance. The background difference method can be taken for vehicle detection. In this section, we select a relatively up to date method of vehicle speed measurement that employs the single-shot detector (SSD) algorithm as a comparison algorithm to complete the vehicle target detection and then uses the optical flow algorithm to achieve the target tracking and vehicle speed calculation.

The KFCM method is a commonly used method for congestion discrimination base on kernel function that detects the roads through the multi-frame fusion of traffic video. The ratio of foreground target pixels to background pixels is calculated to obtain the road space duty cycle. Traffic flow is calculated by the vibe algorithm, and the macro- optical flow speed of the entire lane can be obtained by integrating the Harris corner detection. The cluster center of the traffic state can be found to establish the traffic congestion and obtain the current traffic state by the KFCM algorithm. Another method for congestion discrimination is based on Bayesian decision-making, where the traffic flow and occupancy can be taken as the discriminant parameters and the Bayesian classifier is generated by learning the historical data in the smooth and congestion states to classify the real-time data and identify the traffic state.

### 5.1. Experimental Conditions, Parameters and Evaluation Methods

The experimental simulation in this paper is carried out under the framework of pytorch. An Intel (R) Core i5-3470 processor with a 3.2 GHz clock, 8 Gb memory, and the Ubuntu 16.04 operating system is used in the experiments. The graphics card model is NVIDIA GeForce (R) GTX 1080Ti, and the NVIDIA CUDA9.0 acceleration toolbox is used. The test video selected here is the actual single-channel monitoring video taken by a camera at an intersection in Jingshi Road and Shungeng Road in Jinan City, China, with a duration of 10 min, a resolution of 1280 × 720, and a frame rate of 10.67 fps. Video duration is greater than three semaphore cycles. The experimental parameters used in the network training stage are shown in Table 1.

We get 6450 vehicle target detection data sets by intercepting each frame of the traffic intersection surveillance video and crawling, and then divide the data sets according to the ratio of the training set, test set, and verification set as 6:2:2. The specific division results are shown in Table 2.

The performance of the vehicle target detection algorithm is mainly evaluated by the following indicators.

(1) *Precision* (P). Precision refers to the proportion of the positive samples in the total samples detected by network prediction. The so-called positive samples include two kinds: the real samples (*TP*), that is, the real category is consistent with the detected target category, and the false positive samples (*FP*), that is, the real category is inconsistent with the detected target category. It is denoted as *Precision* = *TP*/(*TP* + *FP*).
(2) *Recall* (R). Recall rate refers to the proportion of the samples correctly predicted in the real samples during network detection. There are two kinds of samples with correct prediction: the real samples mentioned above and the false negative samples (*FN*) whose targets exist and are not detected. We can write it as *Recall* = *TP*/(*TP* + *FN*).
(3) *Average precision* (AP). It is seen that the accuracy rate and recall rate are contradictory to a certain extent, the ideal target detection network usually requires that the recall rate should be improved while the accuracy rate is maintained at a high level. Therefore, the results can be drawn one by one from the beginning according to P and R, and the balance between them can be reflected by an obvious curve which is called precision recall (P-R) curve. The area under the P-R curve and around the coordinate axis is the average accuracy, which can reflect the performance of the target detection network to a certain extent. The expression is $AP=\int_{0}^{1}P(R)dR$.
(4) *Mean average precision* (mAP). The above-mentioned AP is the average precision for a certain type of target, and mAP means the average precision for all the target categories. We can write is as $mAP=\frac{\sum_{i=1}^{N}AP_{i}}{N}$.

At the same time, in the process of congestion identification algorithm, the speed threshold is set to be 30 km/h. When the speed is less than this threshold, we consider that it is congestion state. The algorithm uses a judgment interval of 20 s, and the whole judgment cycle is 160 s.

### 5.2. Experiment Comparison

#### 5.2.1. Comparison of Vehicle Target Detection

The YOLOv3 algorithm for object detection in this paper is compared with the typical SSD algorithm and Faster R-CNN algorithm. In the experiment, the same training set (i.e., the vehicle data set mentioned above) is used to train each algorithm, and the relevant network parameters are set the same above. The simulation results are provided in Table 3.

From the above results, we can see that the Faster R-CNN algorithm is a two-stage target detection algorithm. Although the detection accuracy is ideal, its vehicle speed is seriously restricted, which cannot meet the real-time requirement. The SSD algorithm is faster than the faster R-CNN in terms of detection speed, but its accuracy is slightly lower. Generally, the YOLOv3 algorithm achieves a good trade-off between speed and accuracy, which meets the real-time detection and gets accurate results.

#### 5.2.2. Comparison of Global Speed Detection

Two virtual detection lines with the distance less than the shortest vehicle length are set for the speed detection lane in the test sample video with the detection method based on the double detection lines in [31], and the distance between them is L. The video frame number is recorded as video 1 when the first detection line detects the target vehicle as the vehicle passes through and video 2 when the second detection line detects the target, respectively. The speed is calculated by the speed formula. S.Q. Wu proposed a feature-matching vehicle speed measurement based on SSD in [32], where SSD is first used as the target detection algorithm to get the target vehicle position and then the optical flow method is employed to calculate the vehicle speed. In our experiment, vehicle speed is obtained through manual measurement for the accuracy of the test results, and we consider different weather conditions to make the test results more practical and convincing.

Table 4, Table 5 and Table 6 show that the virtual coil algorithm based on the background difference is slightly more accurate in cloudy and rainy conditions than on sunny case due to the sensitivity of the background difference to the change of light. When the light is strong, the target vehicle will be lost. The speed measurement method based on the double detection is simple in implementation and only calculates the gray level of the video image in the coil. However, its speed measurement error is high and the accuracy is low, which affects the calculation of the global speed. The SSD algorithm has significant improvement in accuracy, due to the insensitivity to light changes and fast operation speed. However, the SSD has a low detection rate for small targets, which influences the accuracy of the global vehicle speed measurement to some extent. The YOLOv3 algorithm is employed to detect the vehicles in this study, and the results show that its accuracy is high and the detection speed is very fast. It also adds a feature pyramid, which can detect the small targets well and directly calculate the speed vector with optical flow method to reduce the error of pixel conversion. Also, various weather conditions have no much impact on the accuracy of the vehicle speed measurement, which shows the robustness of the proposed algorithm for practical use.

#### 5.2.3. Comparison of Traffic State Discrimination

We also compare the KFCM algorithm in [26] and the traffic discrimination based on Bayes in [27] with our algorithm. The traffic road monitoring video dataset is selected from the Computer Vision Laboratory of the University of California at Berkeley to train the KFCM algorithm and Bayesian classifier, which includes different light and weather conditions with complicated scenes. This dataset manually divides the road conditions into three types, smooth, slow driving, and congestion. Among the total 254 videos, 165 are smooth road videos, 45 are slow driving videos, and 44 are congested road videos. Table 7 shows the comparison results.

When the time is at 60 s, the test results of the three methods are smooth and coincide with the actual situation. At 90 s, the green light immediately turns red, and the speed gradually decreases. The results of the KFCM algorithm are smooth because the determination of the initial clustering center and the number of the clusters have a certain impact on the results, resulting in the smooth state is judged as congestion. Around 120 s, this red part is the waiting time. The judging results of the KFCM and Bayesian-based discrimination algorithms are congestion. The introduction of new sample information will cause an interpolation function for the Bayesian-based discrimination algorithm, and serious fluctuation will affect the accuracy of the results.

### 5.3. Analysis of Experimental Results

**Figure 6:**
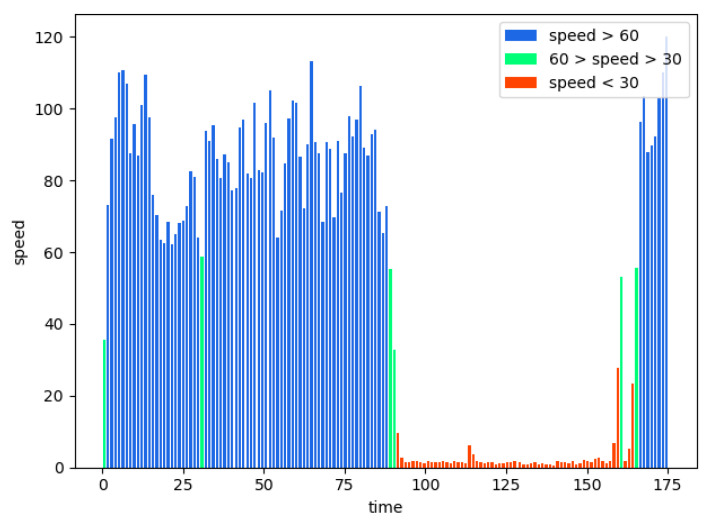
Speed distribution of single signal period.

Figure 6 shows the velocity distribution of the frame rate of the 12 videos measured in one signal cycle with the proposed method. In Figure 6, the signal interval is approximately 160 s, and the global vehicle speed is maintained at a high speed for the first 80 s as the normal traffic condition. The speed is decreased to 0 after 90 s due to a traffic jam or a red light. The congestion identification algorithm is used to establish the average speed *V* at 20 s as a judgment, and the congestion speed threshold is 30 km/h. The maximum number of the congestion judgments *m* mentioned previously should cover three traffic lights. Therefore, we set *m* = 8 times. The speed in the first 80 s is higher than the congestion speed threshold. The traffic state is kept at *S*_0_, and we output the non-congestion signal. The speed in the next 20 s is below the congestion speed threshold and c < 8. Hence, the state changes to *S*_1_, and the output state is slow driving. Then, the algorithm checks the vehicle speed repeatedly until 160 s and the state remains at *S*_1_. Therefore 80–160 s is considered to be red light time and slow driving. The speed in the next 20 s is higher than the congestion speed threshold, and the output state becomes smooth. The experiment results show that we can get the ideal speed by the proposed algorithm and our judgment result is accurate, which verifies the efficiency of the proposed algorithm.

## 6. Conclusions

In this paper, we employ the YOLOv3 algorithm and select the ROI to detect the vehicle targets, and improve the congestion detection speed and accuracy for the urban intersection road. We take the vertex of the detection box as the image feature point and further utilize the LK optical flow method to calculate the global speed. The calculations of the proposed method are significantly reduced compared with the traditional optical flow method. Finally, the global speed experiments and analysis are performed to determine the road traffic conditions, which show that the proposed method has strong anti-interference ability and good vehicle congestion detection accuracy. The method based on YOLOv3 and optical flow method can effectively monitor the intersection congestion conditions in real time and greatly reduce the manual workload.

## Figures and Tables

**Table 1:** Network training parameters.

Parameter Name	Parameter Value
Batch size of input data	6
Resize input image	416 × 416
Sampling step	32
Number of iterations	100
Initial learning rate	0.0001
End of learning rate	0.000001
Momentum parameter	0.9

**Table 2:** Vehicle target detection dataset.

Vehicle	Training Set	Test Set	Validation Set	
6450	3870	1290		1290

**Table 3:** Results of different target detection methods.

Method	mAP@0.5	FPS	Precision (Conf = 0.25)	Recall (Conf = 0.25)
SSD	82.8%	24	79.5%	86.5%
Faster R-CNN	90.1%	6	91.7%	90.0%
YOLOv3	89.7%	44	89.2%	90.1%

**Table 4:** Sunny test results.

Method	Vehicle Detection Accuracy	Vehicle Detection Time	Speed Measurement Accuracy
From virtual detection lines	82.8%	69 ms	79.5%
Speed detection algorithm on the basis of SSD	88.6%	67 ms	85.2%
Proposed Algorithm	89.5%	31 ms	92.2%

**Table 5:** Cloudy test results.

Method	Vehicle Detection Accuracy	Vehicle Detection Time	Speed Measurement Accuracy
From virtual detection lines	84.3%	64 ms	80.1%
Speed detection algorithm on the basis of SSD	88.6%	67 ms	85.5%
Proposed Algorithm	89.5%	31 ms	92.8%

**Table 6:** Rain test results.

Method	Vehicle Detection Accuracy	Vehicle Detection Time	Speed Measurement Accuracy
From virtual detection lines	84.1%	64 ms	79.9%
Speed detection algorithm on the basis of SSD	88.4%	67 ms	85.3%
Proposed Algorithm	89.4%	30 ms	92.4%

**Table 7:** Congestion discrimination by different methods.

Time	KFCM Algorithm	Traffic Discrimination on the Basis of Bayes	Proposed Algorithm
60 s	Smooth	Smooth	Smooth
90 s	Smooth	Slow driving	Slow driving
120 s	congestion	congestion	Slow driving

## Data Availability

The data presented in this study are available on request from the corresponding author. The data are not publicly available due to involve a certain degree of privacy.
